# Madagascine Induces Vasodilatation via Activation of AMPK

**DOI:** 10.3389/fphar.2016.00435

**Published:** 2016-11-25

**Authors:** Dapeng Chen, Bochao Lv, Sei Kobayashi, Yongjian Xiong, Pengyuan Sun, Yuan Lin, Salvatore Genovese, Francesco Epifano, Shanshan Hou, Fusheng Tang, Yunyan Ji, Dandan Yu

**Affiliations:** ^1^Dalian Medical University, DalianChina; ^2^Yamaguchi University School of Medicine, YamaguchiJapan; ^3^Central Laboratory, the First Affiliated Hospital, Dalian Medical UniversityDalian, China; ^4^Dipartimento di Farmacia, Università degli Studi “G. D’Annunzio” Chieti-Pescara, ChietiItaly

**Keywords:** madagascine, AMPK, eNOS, vasospasm, vasoconstriction

## Abstract

Madagascine (3-isopentenyloxyemodin) can be chemically synthesized or purified from several *Rhamnus* species, and it is found to have more potent biological activities than the parent compound emodin. The aim of this study is to characterize the vasodilatory effect of madagascine on vasoconstriction and sphingosylphosphorylcholine induced vasospasm in *ex vivo* and reveal the potential mechanisms *in vitro*. The effects of madagascine on vasoconstriction of rat mesenteric resistance arteries (MRAs) induced by K^+^, methoxamine, and endothelin-1 were, respectively, studied. The cholesterol-enriched porcine coronary vascular smooth muscle (VSM) strips were used to investigate the effects of madagascine on abnormal constriction induced by sphingosylphosphorylcholine (SPC) which has a pivotal role in vasospasm. The vasodilatory effect was induced by madagascine (0.3–100 μM) in isolated rat MRAs and the vasodilatory effect was blocked by NO synthase inhibitor L-NAME and AMPK inhibitor compound C. Madagascine (10 μM) also significantly relaxed the abnormal constriction in porcine VSM induced by SPC and the effect was abolished by compound C. Madagascine significantly increased the phosphorylation of endothelial nitric oxide synthase (eNOS) in endothelial cells while decreasing the phosphorylation of myosin phosphatase target subunit 1 (MYPT1) in VSM cells. Madagascine-induced vasodilatation was abrogated using small interfering RNA knockdown of AMPK. In summary, madagascine exerted vasodilatation through activating AMPK, leading to the activation of eNOS in endothelium and inhibition of ROCK/MYPT1 in VSM. This study suggests the potential value of madagascine in amelioration of vasospasm related cardiovascular diseases.

## Introduction

Vascular vasodilatation is beneficial for amelioration of cardiovascular diseases including essential- and renal-parenchymal-disease-related hypertension, vascular remodeling, cardiac infarction, and congestive heart failure (Hisham and Bayraktutan, 2012; Kumar et al., 2012; Machino et al., 2014). Vascular tone is determined by the vasoconstriction and is regulated by a complex interplay of vasodilator and vasoconstrictor substances (Wang et al., 2011). The drugs which can directly or indirectly induce vascular vasodilatation including inhibitors of renin-angiotensin system, antagonists of adrenergic receptors, diuretics, nitrates, calcium channel blockers. (Kondo et al., 1979; Cox and Rusch, 2002; Ji, 2013; Salihi, 2013) possess the potential in ameliorating cardiovascular diseases.

Recent studies suggest AMP-activated protein kinase (AMPK) is a new therapeutic target for vasodilatation. In endothelial cells, AMPK is activated through the phosphorylation by LKB1 and CAMKK (Stahmann et al., 2006). The activation of AMPK leads to vasodilatation through the phosphorylation of epithelial nitric oxide synthase (eNOS) at site Ser1177 in epithelium and a direct inhibition of vascular smooth muscle (VSM) constriction (Reihill et al., 2007; Bradley et al., 2010; Shuangxi et al., 2011). Smooth muscle constriction is induced by phosphorylation of 20-kDa myosin light chain (MLC), which is regulated by both Ca^2+^ dependent and Ca^2+^ independent mechanisms (Somlyo and Somlyo, 1994). AMPK activation leads to inhibition of Rho-associated protein kinase (ROCK) which mediates Ca^2+^ independent VSM constriction by inhibiting myosin phosphatase via phosphorylation of myosin phosphatase target subunit 1 (MYPT1) (Somlyo, 2002; Shuangxi et al., 2011). Sphingosylphosphorylcholine (SPC) generated by *N*-deacylation of sphingomyelin which is one of the most abundant lipids in cell membrane (Yue et al., 2015). SPC is a phospholipid mediator in blood plasma and it exert multifunctional role in cell physiological regulation (Satoshi et al., 2002). SPC-mediated activation of ROCK has been proved to be involved in pathogenesis of vasospasm (Fumiaki et al., 2002; Somlyo, 2002). However, the relationship and between AMPK activation and SPC induced vasospasm remains unknown.

**FIGURE 1:**
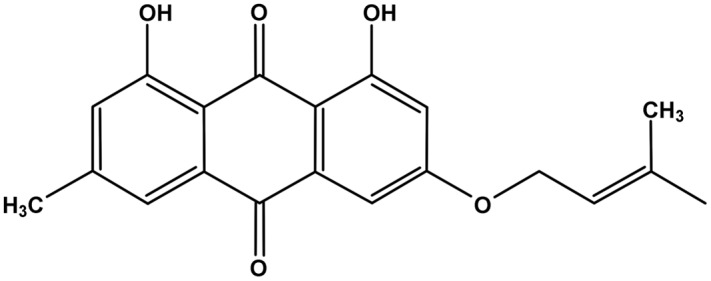
**Chemical structure of madagascine**.

Madagascine (3-isopentenyloxyemodin) is a natural compound containing an anthraquinone core linked to a 3, 3-dimethylallyoxy chain (**Figure 1**). The trivial name of madagascine derives from the natural source, *Harungana madagascariensis* Poir, from which it was isolated and structurally characterized for the first time (Ritchie and Taylor, 1964; Epifano et al., 2013). Subsequently madagascine has also been obtained from several other natural sources including medicinal plants belonging to *Rhamnus* spp. (Delle Monache et al., 1987; Iinuma et al., 1995; Mbaveng et al., 2008). Natural compounds containing an anthracene are widely distributed in the plant kingdom and madagascine emerged as one of the most promising compound from a pharmacological point of view (Epifano et al., 2013). Madagascine have been reported to have multiple biological activities including antioxidant, antimicrobial, and anticancer effects (Mbaveng et al., 2008; Ee et al., 2011; Epifano et al., 2013).

Emodin is found to induce the activation of AMPK in skeletal muscle and liver cells (Song et al., 2013; Subramaniam et al., 2013). Under the same experimental conditions, compared with emodin, the biological activity was more potent and safe (Mbaveng et al., 2008; Ee et al., 2011). Based on our pre-experiments, the present study was designed to characterize the vasodilatory effect of madagascine on vasoconstriction and SPC induced vasospasm in *ex vivo* and *in vitro*. The isolated rat mesenteric resistance arteries (MRAs) were used to investigate the effects of madagascine-mediated activation of AMPK on vasoconstriction. The porcine coronary arteries were used to investigate the effects of madagascine-mediated activation of AMPK on abnormal constriction induced by SPC because porcine coronary arteries are cholesterol-enriched and cholesterol potentiates the Ca^2+^ independent of VSM constriction mediated by SPC (Noriyasu et al., 2006). The vasodilator properties of madagascine were also investigated by using human umbilical vein endothelial cells (HUVECs) and human coronary artery smooth muscle cells (HCASMCs), respectively.

## Materials and Methods

### Animals

The experimental protocol was approved by Dalian Medical University Animal Care and Ethics Committee at June 8th, 2012, and all animals used were maintained in accordance with National Institutes of Health Guide for Care and Use of Laboratory Animals (Publication no. 85-23, revised 1985). Fifty Male Wistar rats (weighing 200–300 g) were obtained from Experimental Animal Center, Dalian Medical University. [Certificate of Conformity: No. SCXK (Liao) 2008-0002]. The animal protocol was designed to minimize pain or discomfort to the animals. The animals were acclimatized to laboratory conditions (23°C, 12 h/12 h light/dark, 50% humidity, *ad libitum* access to food and water) for 2 weeks before experimentation. All rats were euthanized by barbiturate overdose (intravenous injection, 150 mg/kg) for mesenteric arteries isolation. Porcine coronary arteries (20–30 mm from the origin of the proximal portion of left anterior descending arteries) were obtained from a local abattoir.

### Reagents and Cell Lines

Madagascine was supplied by Francesco Epifano and Salvatore Genovese. AMPK-α1+α2 antibodies (ab800039), AMPK-α1 (phospho T183) +α2 (phospho T172) antibodies (ab72845), eNOS antibodies (ab5589), and eNOS (phospho S1177) antibodies (ab184154) were bought from Abcam (Hong Kong) Ltd. (Hong Kong, China). MYPT1 p-MYPT1 (phospho Ser 695) antibodies (sc-33360) were bought from Santa Cruz Biotechnology, Inc. (Santa Cruz, CA, USA). MLC antibodies (3672) and phosphor-MLC (Ser 19) antibodies (3671) were bought form Cell Signaling Technology, Inc. (USA). GAPDH (10494-1-AP) antibodies and MYPT1 antibodies (22117-1-AP) were bought form Proteintech Group, Inc. (Wuhan, China). SPC was bought from Biomol. Unless otherwise indicated, chemicals were obtained from Sigma-Aldrich (St Louis, MO, USA). The cell HUVECs and HCASMCs were obtained from cell bank of Shanghai Institute (Shanghai, China). The cells used in this study were evaluated before experiment including the expression of eNOS and AMPK in these cell lines. No significant inter-species variations in AMPK and eNOS signaling which affect the results in this study were observed according to previous publications and pre-experiments.

### Perfusion of Rat Mesenteric Resistance Arteries

The rat MRAs were isolated and prepared for perfusion according to the methods by Sun et al. (2009). The MRAs were placed in organ bath maintained at 37°C, perfused with a modified Krebs solution [modified Krebs solution in mM: NaCl 119.0, KCl 4.7, CaCl2 2.4, MgSO4 1.2, NaHCO3 25.0, KH2PO4 1.2, EDTA 0.03, and D-glucose 11.1 (pH 7.4)] at a constant flow rate of 5 mL/min with a peristaltic pump (Chengdu TME Technology Co, Ltd, China). Changes in the perfusion pressure were recorded by BL-420F biological system (Chengdu TME Technology Co, Ltd, China) (Chen et al., 2015). The endothelium of the MRAs was removed through the perfusion of 1.80 mg/mL sodium deoxycholate in saline for 30 s as described previously (Shiraki et al., 2000). The MRAs were perfused with 100 mM papaverine (PPV) to induce complete relaxation for confirmation of the vascular activity at the end of each experiment.

### Simultaneous Measurement of [Ca^2+^]i and Force of Porcine VSM *In situ*

Porcine coronary arteries were placed in 4°C physiological salt solution (PSS; in mM: 123 NaCl, 4.7 KCl, 15.5 NaHCO_3_, 1.2 KH2PO_4_, 1.2 MgCl_2_, 1.25 CaCl_2_, and 11.5 D-glucose). The arteries were cut into strips (1 mm × 4 mm) without endothelium and adventitia. These strips were mounted vertically at the organ bath filled with PSS, gassed with 5% CO_2_/95% O_2_, and maintained at 37°C. The isometric force of VSM strips was measured by a force transducer (TB-612T, Nihon Koden). Effect of madagascine on contractile force was investigated at the maximum and steady state of SPC or 40 mM K^+^ induced constriction.

The contractile force and changes in intracellular Ca^2+^ ([Ca^2+^]i) were simultaneously measured using porcine VSM strips (Fumiaki et al., 2002). The VSM strips were loaded with 12.5 μM fura-2/AM. Changes in [Ca^2+^]i were continuously recorded with a spectrofluorometer (CAM-230, Japan Spectroscopic) equipped with a randomized optical fiber system (Todoroki-Ikeda et al., 2000; Noriyasu et al., 2006).

### Western Blot Analysis

Total protein was isolated from HUVECs or HCASMCs. The blots on nitrocellulose filter membrane were probed with corresponding antibodies. The bands were detected and quantified using MultiSpectral imaging system (UVP, Cambridge, UK).

### Cell Transfection

The HUVECs or HCASMCs cells were transfected with Lipofectamine 2000 (Invitrogen) and AMPK-α1/α2-targeted or a control small interfering RNA (siRNA) oligos (Dharmacon, Lafayette, CO, USA) according to the manufacturer’s instructions (Takara Biotechnology (Dalian) CO., LTD). The siRNA sequence was as follows: 50-ACC GAG CUA UGA AGC AGC UGG GUU U-30. The efficiency of gene silencing was confirmed by Western blotting analysis.

### Statistical Analysis

Data analysis was conducted in a blinded manner according to single-blind study design. The One-Way ANOVA was used where three or more groups of data were compared. Data were expressed as the mean ± SD. The data followed a normal distribution and each group had equal variances. To further evaluate the data, Kruskal–Wallis rank sum test was used. All experiments were repeated for at least six times.

## Results

### Effects of Madagascine on the Vasoconstriction of MRAs

**FIGURE 2:**
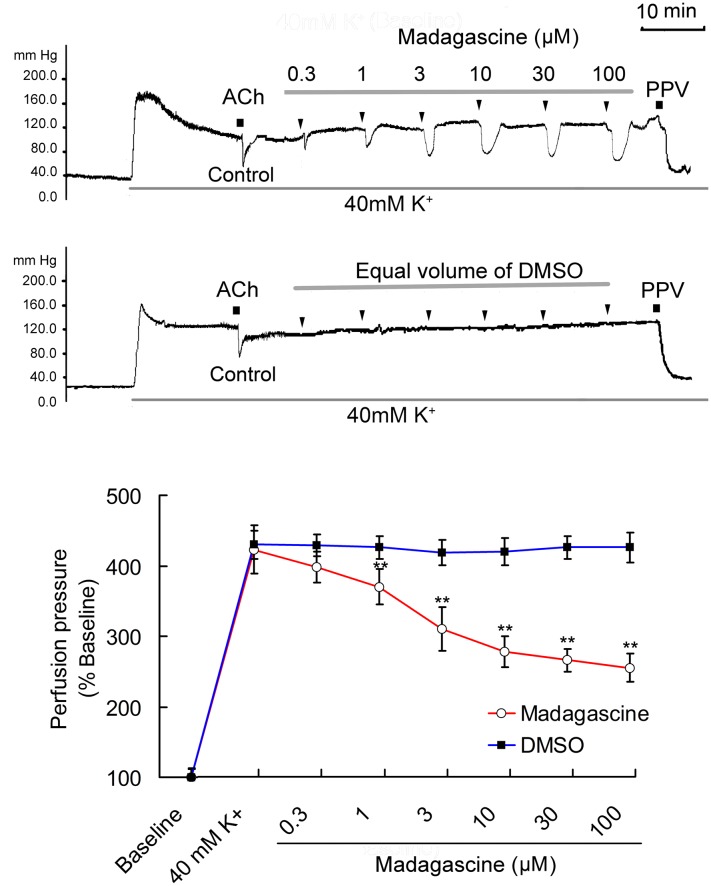
**Madagascine (0.3–100 μM) inhibited the constriction of rat mesenteric resistance arteries (MRAs) induced by 40 mM K^+^.** The doses of madagascine were selected according to pre-experiments. Isolated rat MRAs with intact endothelium were perfused with Krebs solution and the perfusion pressure was stimulated by continuous perfusion of 40 mM K^+^. After the elevated perfusion pressure stabilized, Krebs solution containing 40 mM K^+^ and madagascine at a concentration of 0.3, 1, 3, 10, 30, or 100 μM was perfused, respectively. Acetylcholine (ACh, 1 nM) treatment is a bolus injection. Vasodilatation response to ACh confirms the intact of endothelium. Data are expressed as the mean ± SD and the response to Krebs solution was set as baseline. Other data are the relative values compared with baseline. ^∗∗^*p* < 0.01 compared with 40 mM K^+^, *n* = 6 tissues.

**FIGURE 3:**
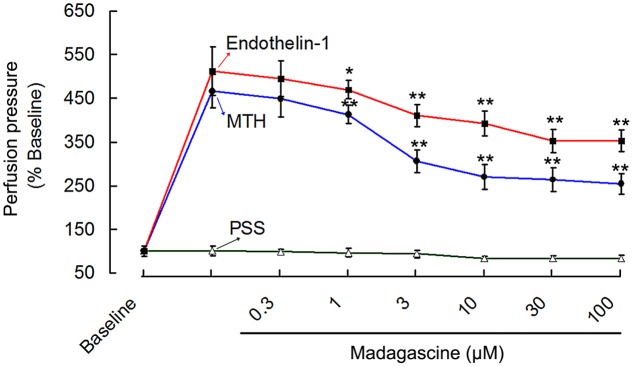
**Madagascine (0.3–100 μM) inhibited the constriction of rat MRAs induced by endothelin-1 (10 nM) and methoxamine (MTH, 7 μM).** The perfusion pressure of isolated rat MRAs with intact endothelium were stimulated by continuous perfusion of endothelin-1/methoxamine, respectively. After the elevated perfusion pressure stabilized, Krebs solution containing endothelin-1/methoxamine and madagascine at a concentration of 0.3, 1, 3, 10, 30, or 100 μM was perfused, respectively. The intact of endothelium was confirmed through vasodilatation response to ACh. Data are expressed as the mean ± SD and the response to Krebs solution was set as baseline. Other data are the relative values compared with baseline. ^∗^*p* < 0.05, ^∗∗^*p* < 0.01 compared with endothelin-1/methoxamine, *n* = 6 tissues.

Based on pre-experiments, 0.3–100 μM madagascine were selected to study its vasodilatory effects. Madagascine reduced constriction of rat isolated MRAs induced by 40 mM K^+^ in a concentration range of 0.3–100 μM. Madagascine-induced vasodilatation was transient and disappeared within about 6 min. Equal volume of vehicle DMSO did not show any inhibitory effect on the constriction of MRAs (**Figure 2**). Madagascine also relaxed MRAs pre-contracted by methoxamine and endothelin-1. However, madagascine did not significantly change the basal tension (**Figure 3**). The rat MRAs remained in good condition after madagascine treatment by washing.

### Effects of L-NAME and Compound C on Madagascine Induced Vasodilatation

**FIGURE 4:**
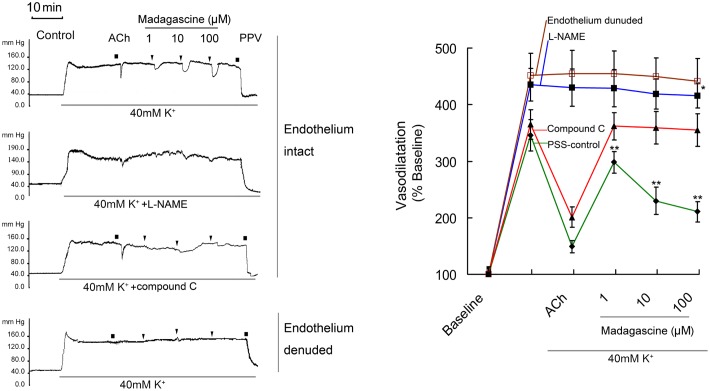
**Effects of compound C and L-NAME on madagascine-exerted vasodilatation.** After 40 mM K^+^ induced high perfusion pressure stabilized, Krebs solution containing 40 mM K^+^ + L-NAME (100 μM) and 40 mM K^+^ + compound C (20 μM) was perfused, respectively. And after the perfusion pressure stabilized, Krebs solution containing 40 mM K^+^ + L-NAME (120 μM) + madagascine and 40 mM K^+^ + compound C (20 μM) + madagascine was perfused. Acetylcholine (ACh, 1 nM) treatment is a bolus injection. Vasodilatation response to ACh confirms the intact of endothelium. Data are expressed as the mean ± SD and the response to Krebs solution was set as baseline. Other data are the relative values compared with baseline. ^∗^*p* < 0.05 compared with stable elevated perfusion pressure by Krebs solution containing both 40 mM K^+^ and L-NAME, *n* = 6 tissues; ^∗∗^*p* < 0.01 compared with stable elevated perfusion pressure by Krebs solution containing 40 mM K^+^ (PSS control), *n* = 6 tissues.

**FIGURE 5:**
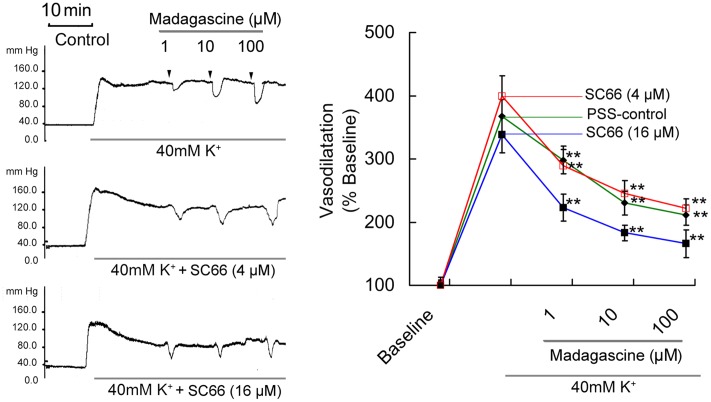
**Effects of AKT inhibitor SC66 on madagascine-exerted vasodilatation.** After 40 mM K^+^ induced high perfusion pressure stabilized, Krebs solution containing 40 mM K^+^ + SC66 (4 μM) and 40 mM K^+^ + SC66 (16 μM) was perfused, respectively. And after the perfusion pressure stabilized, Krebs solution containing 40 mM K^+^+ SC66 (4 μM) + madagascine and 40 mM K^+^ + SC66 (16 μM) + madagascine was perfused. Data are expressed as the mean ± SD and the response to Krebs solution was set as baseline. Other data are the relative values compared with baseline. ^∗∗^*p* < 0.01 compared with stable elevated perfusion pressure by Krebs solution containing 40 mM K^+^ and SC66, *n* = 6 tissues.

Madagascine did not induced vasodilatation on 40 mM K^+^-induced constriction of rat MRAs without epithelium (**Figure 4**). In MRAs with an intact endothelium, madagascine exerted vasodilatation effect was significantly blocked by NO synthase inhibitor L-NAME and AMPK inhibitor compound C, respectively. These results suggest that AMPK/eNOS signaling pathway is involved in madagascine-induced vasodilatation (**Figure 4**). Both AMPK/eNOS and AMPK/AKT/eNOS are known to be related to increase the production of NO (Bradley et al., 2010; Wei et al., 2016). As shown in **Figure 5**, madagascine-induced vasodilatation in the presence of SC66 was more transient than that in the absence of SC66. However, in this study, AKT inhibitor did not significantly block madagascine induced vasodilatation rate (**Figure 5**). The potential role of AMPK/eNOS pathway was mainly studied in this study.

### Effects of Madagascine on AMPK and eNOS in HUVECs

**FIGURE 6:**
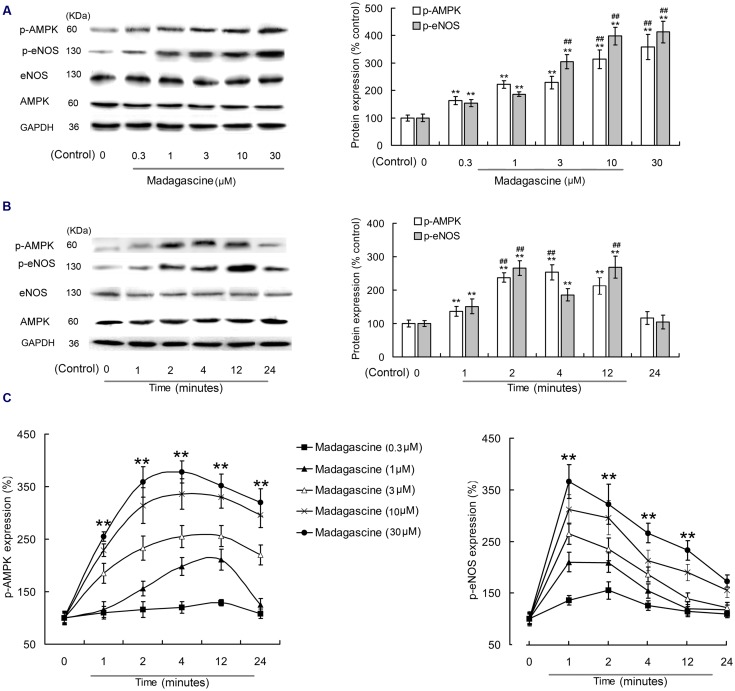
**Effects of madagascine on AMPK phosphorylation in human umbilical vein endothelial cells.** **(A)** 0.3–30 μM madagascine increased the phosphorylation of eNOS and the phosphorylation of AMPK with incubation time of 4 min. **(B)** 10 μM madagascine increased the phosphorylation of eNOS and the phosphorylation of AMPK with incubation time of 1–24 min. **(C)** Madagascine in different concentrations increased the phosphorylation of eNOS and the phosphorylation of AMPK with different incubation times. Data are expressed as the mean ± SD and the data. The group without madagascine incubation is set to a relative value of 100% (control). Other data are the relative values compared with control. ^∗∗^*p* < 0.01 compared with the control, *n* = 6 tissues; ^##^*p* < 0.01 compared with the corresponding data in 0.3 μM madagascine treated group or 1 min treated group.

**FIGURE 7:**
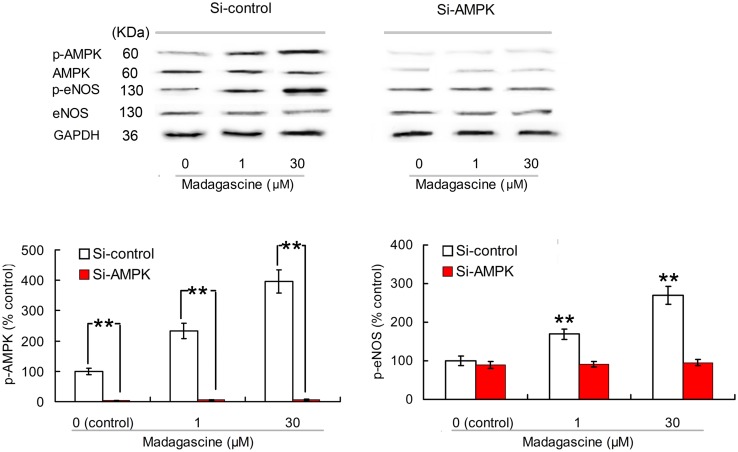
**Effects of small interfering RNA knockdown of AMPK on madagascine induced phosphorylation of eNOS (p-eNOS) in human umbilical vein endothelial cells.** Data are expressed as the mean ± SD and the data observed from madagascine untreated si-control group is set to a relative control of 100%. Other data are the relative values compared with control. ^∗∗^*p* < 0.01 compared with control or as indicated, *n* = 5 samples.

To validate the involvement of AMPK/eNOS signaling pathway in madagascine exerted vasodilatation, the effects of madagascine on AMPK and eNOS were studied using HUVECs. Madagascine, in a concentration range of 0.3–30 μM, induced the phosphorylation of eNOS and the phosphorylation of AMPK, respectively, in a time dependent manner from 1 to 24 min (**Figure 6**). The effects of AMPK knockdown by siRNA in HUVECs were confirmed by Western blotting analysis (**Figures 7A,B**). After siRNA knockdown of AMPK, the madagascine-induced phosphorylation of eNOS was abrogated, indicating that madagascine-induced phosphorylation of AMPK activated the phosphorylation of eNOS, leading to nitrite-mediated blood vessel relaxation (**Figure 7**).

### Effects of Madagascine on SPC Induced Abnormal Constriction

Madagascine did not affect 40 mM K^+^-induced constriction in rat MRAs without epithelium, suggesting that madagascine-activated AMPK induced vasodilatation of VSM is Ca^2+^-independent, because K^+^ depolarization induced vasoconstriction is a typical Ca^2+^ dependent constriction (Satoshi et al., 2002; Somlyo, 2002).

**FIGURE 8:**
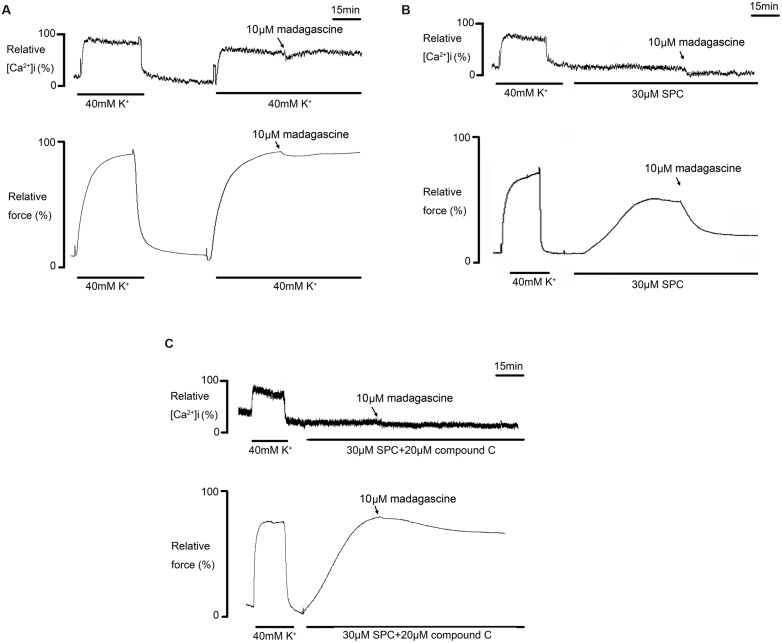
**Effects of madagascine on [Ca^2+^]i and force of porcine vascular smooth muscle (VSM) constriction induced by sphingosylphosphorylcholine (SPC).** **(A)** Effects of madagascine on [Ca^2+^]i and force of porcine VSM constriction induced by 40 mM K^+^; **(B)** Effects of madagascine on [Ca^2+^]i and force of porcine VSM constriction induced by SPC; **(C)** Effects of compound C on madagascine induced modulation. Force and fluorescence ratios were expressed as a percentage and the response to 40 mM K^+^ was set to 100%. Every experiment was repeated at least five times.

As SPC is found to induce Ca^2+^ independent vasoconstriction through activation of ROCK which is involved in hypertension and vasospasm (Satoshi et al., 2002), the effects of madagascine on vasospasm induced by SPC were investigated. The porcine coronary arteries were used here because porcine coronary arteries are cholesterol-enriched and cholesterol potentiates the Ca^2+^ independent of VSM constriction mediated by SPC as described above. As shown in **Figure 8A**, madagascine affects neither the increase of [Ca^2+^]i nor the constriction of VSM induced by 40 mM K^+^. However, SPC induced VSM constriction was significantly reduced by madagascine without significant affecting the slightly elevated [Ca^2+^]i (**Figure 8B**); and madagascine induced vasodilatation was abolished by AMPK inhibitor compound C (**Figure 8C**), indicating that madagascine reduced SPC-induced Ca^2+^ independent vasoconstriction via activating AMPK.

### Effects of Madagascine on Ampk, Mypt1, and Mlc20 in Hcasmcs

**FIGURE 9:**
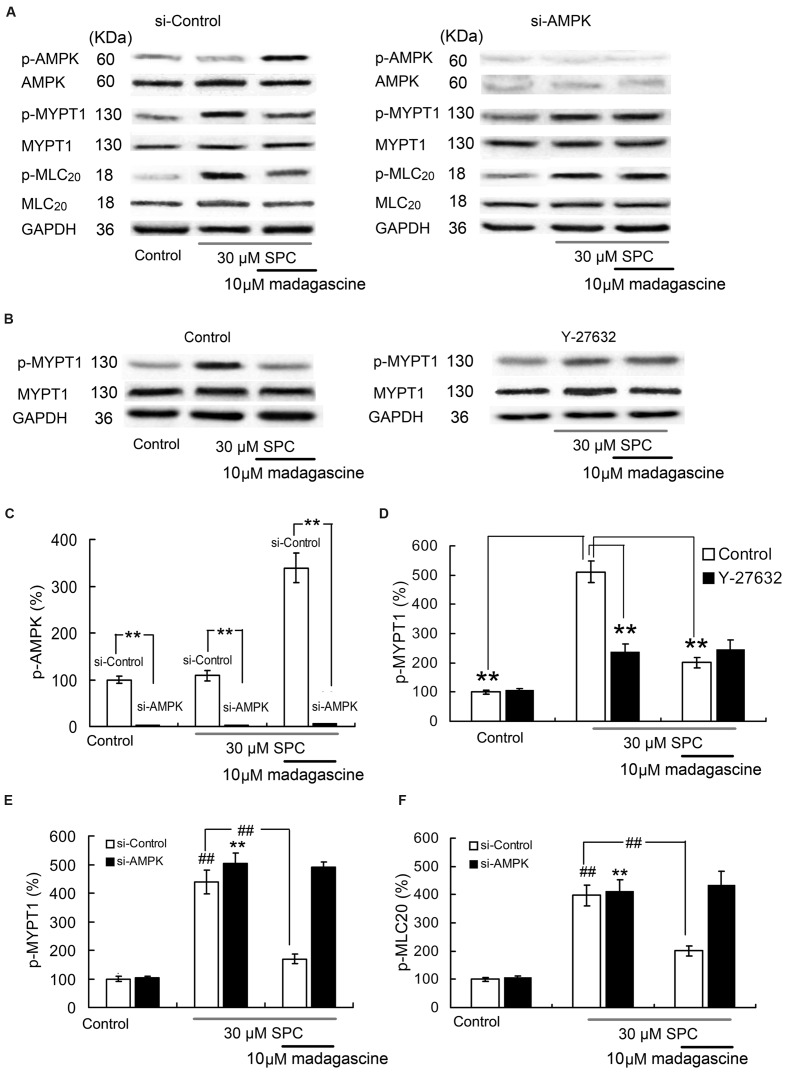
**Effects of small interfering RNA (siRNA) knockdown of AMPK and ROCK inhibitor Y27632 (10 μM) on madagascine induced the phosphorylation of MYPT1 (p-MYPT1) and the phosphorylation of MLC20 (p-MLC_20_) in human coronary artery smooth muscle cells.** **(A)**, **(C)**, **(E)**, and **(F)**: Effects of siRNA knockdown of AMPK on madagascine induced the phosphorylation of AMPK (p-AMPK), p-MYPT1 and p-MLC20. **(B)** and **(D)**: Effects of Y27632 on madagascine induced p-MYPT1. Data are expressed as the mean ± SD and the data obtained from madagascine untreated si-control group is set to a relative value of 100% (control). Other data are the relative values compared with control. ^∗∗^*p* < 0.01 compared with madagascine untreated si-AMPK group or as indicated, *n* = 6 samples; ^##^*p* < 0.01 compared with data obtained from madagascine untreated si-control group or as indicated, *n* = 6 samples.

To validate the involvement of AMPK in madagascine-reduced abnormal constriction induced by SPC, the effects of madagascine on the phosphorylation of MYPT1 and phosphorylation of MLC_20_ in VSM cells with siRNA knockdown of AMPK were studied. The effects of AMPK knockdown by siRNA in HCASMCs were confirmed by Western blotting analysis (**Figures 9A,C**). The phosphorylation of MYPT1 and the phosphorylation of MLC_20_ were significantly increased in HCASMCs incubated with SPC (**Figures 9A,E,F**). Madagascine significantly decreased both SPC-elevated phosphorylation of MYPT1 and the phosphorylation of MLC_20_. The inhibitory effects of madagascine on the phosphorylation of MYPT1 and the phosphorylation of MLC_20_ were abrogated by siRNA knockdown of AMPK (**Figures 9A,E,F**), indicating that madagascine-induced phosphorylation of AMPK inhibited the activity of ROCK (the expression of phosphorylate MYPT1 represents the ROCK activity), leading to the vasodilatation of Ca^2+^-independent vasoconstriction. In order to prevent the false-positive results by ROCK independent phosphorylation of MYPT1 (Kaplun et al., 2003), ROCK inhibitor Y-27632 was also used. As shown in **Figures 9B,D**, the inhibitory effects of madagascine on the phosphorylation of MYPT1 were also significantly abrogated by Y-27632, which confirms that madagascine induced activation of AMPK leads to the inhibition of ROCK.

## Discussion

**FIGURE 10:**
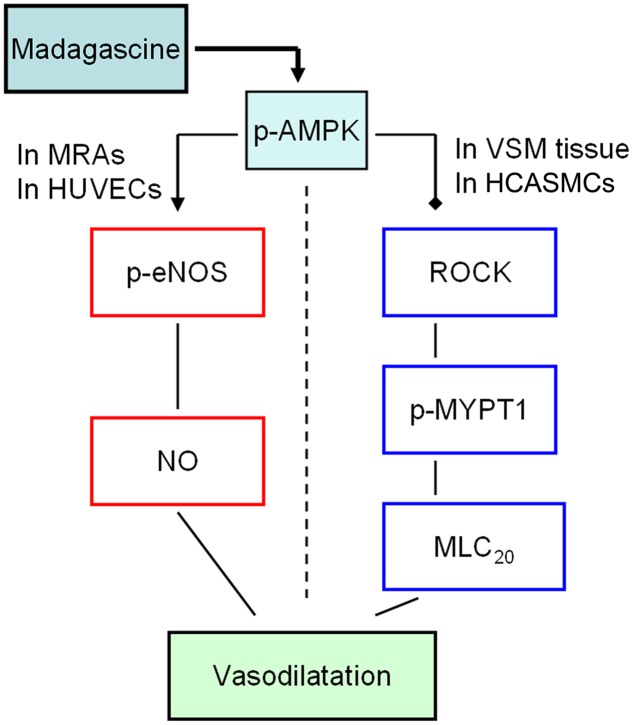
**The potential mechanisms involved in madagascine-activated AMPK induced vasodilatation.** The solid black squares represent for inhibitory effect; the arrow represents for stimulatory effect. MRAs, mesenteric resistance arteries; VSM, vascular smooth muscle; HUVECs, human umbilical vein endothelial cells; HCASMCs, human coronary artery smooth muscle cells.

In this study, madagascine exerted vasodilatory effects in rat MRAs and porcine VSM strips, respectively. The phosphorylation of AMPK was significantly increased by madagascine in a concentration and time dependent manner. The phosphorylation of eNOS in HUVECs was significantly increased and the phosphorylation of MYPT1 in HCASMC cells was significantly decreased by madagascine. These results suggest that AMPK-mediated activation of eNOS in epithelium and inhibition of ROCK/MYPT1 in VSM were involved in madagascine-induced vasodilatation (**Figure 10**).

In the vasculature, the activation of endothelial AMPK has been shown to phosphorylate eNOS at Ser^1177^ and Ser^633^, stimulating NO release and subsequent vasodilatation of blood vessels (Majithiya and Balaraman, 2006). Purified AMPK is also reported to phosphorylate eNOS at Thr^495^ *in vitro* (Mariman and Wang, 2010). In this study, we also found that in the presence of AKT inhibitor SC66, madagascine induced vasodilatation was more transient than that in the absence of SC66. This suggests that the AMPK/AKT/eNOS pathway may also be involved in madagascine induced vasodilatation. AKT pathway may attribute to increase madagascine induced vasodilatation time. However, how can AKT modulate madagascine induced madagascine needs further study.

In this study, madagascine inhibited 40 mM K^+^-induced MRAs constriction and the inhibition was blocked by AMPK inhibitor compound C and eNOS inhibitor L-NAME. These results suggested that AMPK activation in epithelium is involved in madagascine-induced MRAs vasodilatation.

AMPK-mediated relaxation of VSM constriction is also observed where the endothelium is damaged (Majithiya and Balaraman, 2006), suggesting that AMPK activation mediated vasodilatation is not dependent on endothelium. AMPK activation can lead to Ca^2+^ independent vasoconstriction (Somlyo, 2002; Shuangxi et al., 2011) and this effect was also confirmed in the present study as madagascine did not show any relaxant effects on the constriction of MRAs without endothelium in the presence of 40 mM K^+^ (data not shown). Madagascine significantly inhibited the VSM constriction induced by SPC and the inhibition was not abolished by compound C, suggesting that AMPK activation is involved in madagascine induced VSM relaxation. In HCASMC cells, SPC-induced increase in the phosphorylation of MYPT1 and the phosphorylation of MLC_20_ were significantly reversed by madagascine. AMPK knock down block madagascine-induced the inhibition of the phosphorylation of MYPT1 (represents for the ROCK activity) and the phosphorylation of MLC_20_. The results suggest that madagascine-induced AMPK activation is beneficial for amelioration of vasospasm as SPC-mediated activation of ROCK is found to be involved in pathogenesis of vasospasm (Fumiaki et al., 2002; Somlyo, 2002). Although it is applicable to test the drug effects on AMPK or eNOS using HUVEC and HCASMCs (Fumiaki et al., 2002; Satoshi et al., 2002; Noriyasu et al., 2006; Yang et al., 2009; Xu et al., 2012), it is better to use primary cells to study. Due to limited conditions, these experiments will also be carried out in future study.

This study studied the effects of madagascine on both endothelium and VSM because endothelium and VSM play different pathophysiological roles in vascular diseases (Santulli et al., 2011, 2014). For example, the lack of discrimination between proliferating VSM cells and endothelial cells may increase the risk of late thrombosis following angioplasty (Santulli et al., 2014). Madagascine-induced vasodilatation was transient and this transient vasodilatation effect on blood pressure *in vivo* needs further study. The highest concentration of madagascine used in this study is 100 μM and it was shown that madagascine and its parent compound emodin shows almost no cell toxicity on normal cells but cancer cells (Epifano et al., 2013; Wei et al., 2013). The rat MRAs kept good condition after madagascine treatment by washing in this study, which also confirmed almost no cell toxicity of madagascine. However, the higher concentration of madagascine is also to be used to test its effects *in vivo* study. The aim of this study is to study the vasodilatory effect of madagascine on vasoconstriction *in vitro*, and to uncover weather AMPK activation is involved in madagascine induced vasodilatation. The other roles including potassium channel, alpha adrenergic receptors, and some vasodilatation factors in madagascine induced vasodilatation maybe, respectively, studied in future.

Cardiovascular diseases are often associated with co-occurring atherosclerosis, hypercholesterolemia, and inflammation, and madagascine induced activation of AMPK also is potentially beneficial for the prevention against and amelioration of the co-occurring disorders based on its modulation on VSM and circadian rhythm (Fan et al., 2011). Madagascine has potent biological activity and is safer as a potent AMPK activator. Madagascine-induced activation of AMPK leads to inhibition of vasoconstriction suggests its potential value in amelioration of vasospasm related cardiovascular diseases.

## Author Contributions

YL, DC, FE, SK, and PS designed the study. DC, BL, PS, SH, and DY performed the tissue research. BL, YX, YJ, and FT performed the cell research. YL, SG, and FE contributed reagents or analytic tools. DC, SK, and YX analyzed data. YL and DC wrote and revised the manuscript.

## Conflict of Interest Statement

The authors declare that the research was conducted in the absence of any commercial or financial relationships that could be construed as a potential conflict of interest.
